# Auditory stimulation improves motor function and caretaker burden in children with cerebral palsy- A randomized double blind study

**DOI:** 10.1371/journal.pone.0208792

**Published:** 2018-12-13

**Authors:** Hilla Ben-Pazi, Adi Aran, Anand Pandyan, Nava Gelkop, Gary Ginsberg, Yehuda Pollak, Debby Elnatan

**Affiliations:** 1 Neuropediatric Unit, Shaare Zedek Medical Center, Jerusalem, Israel; 2 School of Health and Rehabilitation, Keele University, Keele, United Kingdom; 3 Physical therapy, Keren-Or Center, Jerusalem, Israel; 4 Meshi Children's Rehabilitation Center, Jerusalem, Israel; 5 Health Economic Consultant, Jerusalem, Israel; 6 The Seymour Fox School of Education, The Hebrew University of Jerusalem, Jerusalem, Israel; Boston Children's Hospital / Harvard Medical School, UNITED STATES

## Abstract

**Aim:**

To investigate the impact of auditory stimulation on motor function in children with cerebral palsy (CP) and disabling hypertonia.

**Method:**

9 matched pairs (age: 7y5m, *SD* 4y1m; 13 boys; gross-motor-functional-classification-scale: median 4; manual-ability-classification-system: median 4) were randomized to receive either auditory stimulation embedded in music (study, *n =* 9) or music alone (sham, control, *n =* 9) for at least 10 minutes 4 times a week for 4 weeks. Goal-Attainment-Scale, Care-and-Comfort-Hypertonicity-Questionnaire, Gross-Motor-Function–Measure and Quality-of-Upper-Extremity-Skills-Test (QUEST) were assessed before and 5 months following intervention.

**Result:**

Children receiving auditory stimulation attained more goals than children who listened to music alone (*p = 0*.*002)*. Parents reported improved care and comfort in children in the study group compared to a slight deterioration in controls (*p* = 0.002). Upper extremity skills improved in the study group compared to controls (*p =* 0.006). Similar gross motor function changes were documented in both groups (*p =* 0.41).

One participant reported increased seizure frequency; no other participants with epilepsy reported increased seizure frequency (*n =* 6/18) and no other adverse events were reported.

**Interpretation:**

Auditory stimulation alleviated hypertonia and improved fine and gross motor functions.

## Introduction

Cerebral palsy (CP), the most common cause of disability in children (~2:1000 live births), is characterized by motor dysfunction and hypertonia (abnormal elevated tone)[1]. Treatments for CP are directed at maximizing motor abilities and improving goal directed function.[2] Even a slight improvement in motor function may have a significant impact on the child and caregivers comfort and quality of life. Non-invasive therapies, medication and surgeries have been tried with variable effectiveness and efficacy.[3] Non invasive treatments are normally preferred due to the potential of having minimal adverse effect.

Transcranial-Magnetic-Stimulation (TMS),[4] Transcranial Direct Current Stimulation (tDCS), [5] and Deep-Brain-Stimulation (DBS),[6] have been shown to improve motor function in children with CP, some with long term effects.[7] The effect of these interventions can, in part, be attributed to the facilitation of plasticity by both direct and indirect modulation of cortical activity. There is evidence that non-invasive low frequency acoustic signals, up to 30 Hz, have been shown to modulate cortical activity.[8]

We hypothesized that the auditory stimulation facilitates cortical plasticity leading to improved motor function in children with CP. In a short term open label pilot study on 4 children with spastic cerebral palsy, we found that children were more relaxed after the intervention. Reports demonstrated improvements in caretaker burden and dissociated movement (QUEST, Elnatan and Gelkop—unpublished data). Following up on these findings, we conducted a randomized-controlled double blind study testing the impact of auditory stimulation on daily motor function and caretaker burden in children with CP.

## Method

### Study design

**Fig 1:**
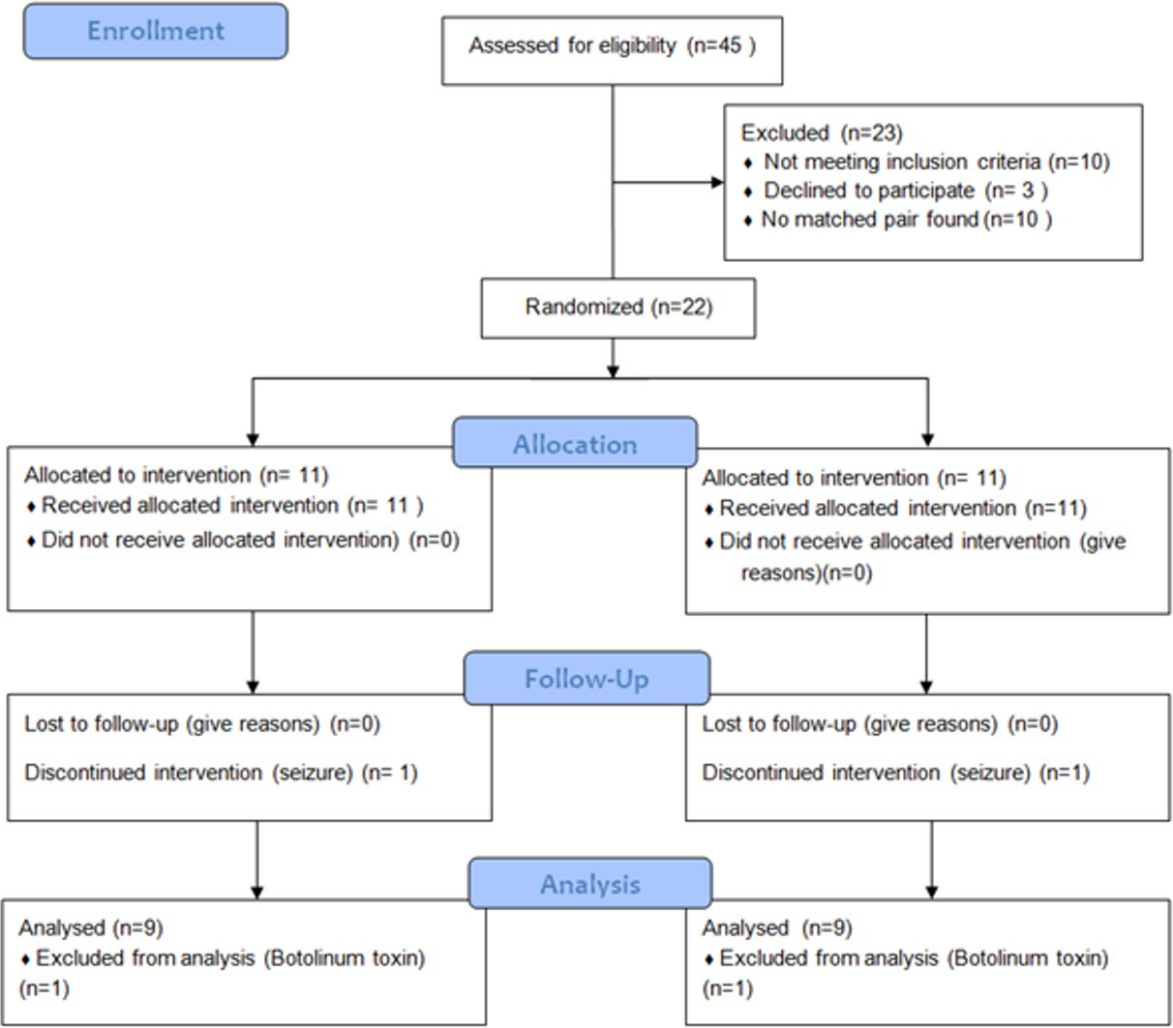
Flow diagram.

A matched pair double blind randomized control study (Fig 1). All children, parents and assessors were blind to group allocation.

### Participants

We enrolled 11pairs of children with cerebral palsy in the study (*n =* 22, age range 2yr 5mo to 17 years, mean age 7yr11m, *SD* 3y11m; 16 boys, 6 girls; Table 1). We recruited participants from three special rehabilitation educational facilities in central Israel according to the following inclusion criteria: (1) age 2–18 years (2) hypertonia interfering with daily functions, (3) ability to use headphones for at least 10 minutes. Exclusion criteria were: 1- a discernible hearing deficit (*n =* 0); 2-no matched pair (*n =* 10); 3-potential/ history of non-compliance as assessed by the child's therapists (*n =* 4); 4- contaminant treatment with medications to reduce hypertonia (*n =* 0); 5- Orthopedic surgery the past year (*n =* 5); 6- Other interventions effecting motor abilities planned during the study period (Botulinum toxin interventions, casting, and surgery; *n =* 4). The Helsinki committee at Shaare Zedek Medical Center approved the study (clinicaltrials.gov ID # NCT00394641). We obtained a written informed consent from all parents of participants.

**Table 1:** Clinical characteristics.

	School	Group	age (Y)	Sex	Comm.	Cog. level	Anatomic distribution	Movement Disorder	GMFCS	MACS	GFMF	QUEST	Care & Comfort
**1**	A	Study	7.2	M	nonV	Severe ID	Quad	D	5	5	3.35	-4.48	2.98
**2**	A	Cont	6.6	M	nonV	Borderline	Quad	S,D	5	5	19.29	2.01	3.22
**3**	A	Study	3.2	M	V	Normal	Di	S	2.5	1	68.63	89.72	2.58
**4**	A	Cont	3.3	M	V	Normal	Di	S	2	1	86.70	87.40	1.48
**5**	A	Cont	2.4	F	nonV	Normal	Quad	S,D	4	3	14.24	17.28	3.13
**6**	A	Study	2.9	M	V	Normal	Quad	S,D	4	3	26.47	43.91	4.50
**7**	B	Study	17	M	V	Borderline	Quad	S,D	5	4	28.57	14.19	3.48
**8**	B	Cont	16	M	V	Borderline	Quad	S,D	5	4	25.51	20.48	3.66
**9**	B	Study	7.5	M	V	Borderline	Di	S	3	3	69.20	70.91	1.71
**10**	B	Cont	8	M	V	Borderline	Quad	S	3	3	66.00	72.54	3.60
**11**	C	Study	5.3	F	nonV	Bellow avg	Quad	S,D	5	5	15.22	0.30	4.99
**12**	C	Cont	6.3	F	nonV	Bellow avg	Quad	S,D	5	4	17.80	21.18	2.80
**13**	C	Study	5.7	M	nonV	Severe ID	Quad	S,D	5	5	17.63	-0.23	4.59
**14**	C	Cont	4.7	M	nonV	Severe ID	Quad	S,D	5	5	12.02	0.06	5.50
**15**	C	Study	10	M	V	Normal	Di	S	4	4	25.82	14.41	4.04
**16**	C	Cont	9.1	F	nonV	Normal	Di	S,D	4	4	33.22	27.66	2.72
**17**	C	Cont	8	F	V	Low	Hemi	S,D	3	3	37.32	47.23	3.19
**18**	C	Study	11	M	V	Low	Hemi	S	3	3	56.84	35.63	3.54

CCHQ = Care and Comfort Hypertonicity Questionnaire; GMFCS = Gross motor functional classification scale; GMFM = Gross Motor Function Measure; MACS = Manual Ability Classification System; QUEST = Quest-Quality of upper extremity skills test; Cont = control; M = male, F = female, V = verbal, nonV = non-Verbal, |Quad = quadriplegia, Di = Diplegia, Hemi = hemiplegia.

The children were paired according to age, gender and CP classification parameters: gross-motor-functional-classification-scale (GMFCS), manual-ability-classification-system (MACS), anatomical distribution (quadriplegia, diplegia, hemiplegia), communication abilities (verbal/ non-verbal) and cognitive abilities (assessed by the teacher from severe cognitive impairment to no cognitive impairment). Random assignment was used to allocate children to either the treatment or control group. The names of two children, comprising a matched pair, were each written on a piece of paper and put in a box. AA randomly picked out a paper from the box. This child was allocated to the treatment group while the second child was allocated to the control group.

### Auditory intervention

We requested that the children would listen to the audio stimulation for at least 10 minutes each session preferably 30 minutes four times a week for 4 weeks. We provided each child with a CD according to her/his group allocation control or treatment. Each child was given a disc with 4–5 audio stimulation tracks, each track including music or nature sounds in the background.

#### Study intervention

Sound frequencies in the gamma range modulated in frequency and/or amplitude according to a fixed protocol. These sounds were embedded in background music or nature sounds according to the child's preferences. The background music was different for each child and was chosen by the child or by the parents (for the younger children). This was done in order to assure that the child would actively listen to the tracks. The gamma tones gradually fade in to the background music/ sounds over the first two minutes.

#### Control conditions

Music and nature sound according to the child’s preference.

We supplied each child with headphones, CD player, diary, and guidance as to how to improve headphone wearing compliance. We instructed participants to listen to the CD through the headphones at least three times a week with no limits on the number of sessions. We asked the caretakers to keep a session log (dates and duration) and to comment (free text) in patient diaries. The children in both groups received the same routine therapy throughout the study.

### Outcome measures

The study took place at home or school according to parents' preference. All the group allocation assessors, caretakers and participants were blinded to group allocation. A repeated measure design to minimize variability was used with repeated assessments—twice over a two-week period at the same time during the day. Baseline assessment was performed during the two weeks prior to the study onset. Post -intervention assessments took place at 20 weeks in order to determine if there are significant benefits to the audio stimulation in the long term.

#### Care and Comfort Hypertonicity Questionnaire (CCHQ)

CCHQ was developed to document the efficacy of interventions in non-functional children with CP (GMFCS levels IV, and V).[9] Items are rated on a 7-point scale from 1 (very easy) to 7 (impossible), depending on the caregiver’s or patients ease or difficulty in performing the task. Score ranges from 1 to 7 with lower scores representing easier care, increased patient comfort, improved communication, self-feeding and play or reduced drooling.[10]

#### Goal Attainment Scale (GAS)

GAS a sensitive individualized, criterion-referenced method measuring specific outcomes on individual goals after a period of treatment and widely used to supplement standardized measures of outcome.[11] For each goal outcome rated by the physical therapist in numerical values from -2 (being the least favorable outcome, much less than expected) to +2 (being the most favorable outcome, more than expected) with a 0 indicating the expected outcome.[12] A scale was calculated based on the percentage of possible improvement attained (e.g. if there were 2 goal outcomes (with a baseline score of -2) that attained scores (on a -2 scale to +2) of +1 and +2 after five months, then the potential improvement attained = 87.5% (i.e. (+3+4)/(maximum possible +8)).

In addition, a GAS T-Score, measuring the achieved change (based on both the physical and occupational therapists readings), was calculated for each child, representing the composite GAS score transformed to a standardized measure using parametric techniques.[13] This transformation provides a numerical T-score which is normally distributed about a mean of 50 (if the goals are achieved precisely) with a standard deviation of around this mean of 10 (if the goals are overachieved or underachieved).[14]

#### Gross Motor Function Measure (GMFM-88)

GMFM a clinical observational tool for evaluating change in gross motor function over time in children with CP.[15] 88-items grouped into 5 functional dimensions: lying & rolling, sitting, crawling & kneeling, standing, and walking, running & jumping. Standardized score is average and ranges from (1 = poor to100 = best performance).[16]

#### Quality of upper extremity skills test (QUEST)

QUEST evaluation of the quality of upper function in four domains: associated movements, grasp, protective extension, and weight bearing. Scores in each domain are calculated in percentages. Standardized score ranging from 1 to 100 with higher scores represent better quality of movement.[17]

### Adverse effects

We asked the parents to report about their children’s bowel and bladder control (5-point scale), sleep, drooling and head control (4-point scale) and constipation (3-point scale; S1 Questionnaire) at baseline and after 5 months.

### Statistical methods

Statistical analyses were performed using SPSS 22.0 (Armonk, NY: IBM Corp). We calculated the intervention efficacy using the improvement of the outcome measures of the study group compared to their matched pair using the Wilcoxon-signed ranked test. Study and Control groups were compared depending on data type with the non-parametric Wilcoxon Signed Ranks test or Fisher’s exact test when relevant. Correlation between variables was assessed with the non-parametric Spearman’s rank order and Pearson’s correlation coefficients.

A p-value of 0.05 or lower was considered statistically significant. Power analysis revealed that such sample size could identify a difference of 0.85 standard deviations, with significance level of 0.05 and power of 0.8. We calculated the sample size at 10 matched pairs in advance. The study was intensive and it took three years to complete the eleven pairs. One pair dropped out and the second was removed when Botox was given unexpectedly to one of the children.

## Results

Eighteen children completed the study (age 7y5mo, *SD* 4y1mo, 13:5, M:F, Table 1 and S1 Table). One pair dropped out since the parents of one of them reported seizures aggravation at the 2nd week. Another pair dropped out since the study participant was treated with botulinum toxin injections within the study period (3 months).

### Baseline assessments

The children had variable motor abilities GMFCS level range 2 to 5 (median 4), MACS level ranging from 1–5 (median 4). Most children had quadriplegia (*n =* 11) followed by diplegia (*n =* 5) and hemiplegia (*n =* 2). Six children had spasticity, one with dystonia and 11 had a mixed movement disorder. Most (*n =* 10/18) children were verbal and some (8/18) were non-verbal). Cognitive abilities classified by the teacher based on formal psychological evaluations and speech assessments, ranging from 1(low) to 3 (normal range), were lower than typically developed children (median 2.5). There were no differences between the study and control groups in age *(*Wilcoxon Signed Ranks *test*, *p =* 0.153),gender (2- sided Fisher's exact test, *p =* 0.294), school allocation (2- sided Fisher's exact test, *p =* 1.000), verbal abilities (2- sided Fisher's exact test; *p =* 0.637), cognitive level *(*Wilcoxon Signed Ranks test, *p =* 0.317), anatomic distribution (Fisher exact test, *p =* 1.000), spasticity (2- sided Fisher's exact test, *p =* 1.000), dystonia (2- sided Fisher's exact test; *p =* 0.620), GMFCS (Wilcoxon Signed Ranks test, *p =* 0.317), MACS (Wilcoxon Signed Ranks test, *p =* 0.317), baseline GFMF (Wilcoxon Signed Ranks test, *p =* 0.859), baseline QUEST (Wilcoxon Signed Ranks test, *p =* 0.214) or baseline CCHQ (Wilcoxon Signed Ranks, *p =* 0.374).

### Outcome measures

There was a vast improvement in function in individual children especially in walking, standing, Care and Comfort (CCHQ), Goal Attainment Scale (GAS) and Quality-of-Upper-Extremity-Skills-Test (QUEST; Table 2).

**Table 2:** Scores at baseline and 5 months for study and control groups.

	Treatment			Controls				
	Before	After		Before	After		p value	
QUEST	29.4	34.2	**4.8**	32.9	31.8	**-1.0**	0.006	sig
Disassociated movement	49.8	53.8	**4.0**	50.7	50.6	**-0.1**	0.39	
Grasp	12.7	17.2	**4.5**	20.2	20.6	**0.4**	0.014	sig
Weight bearing	34.7	37.3	**2.7**	38.7	37.6	**-1.1**	0.014	sig
Protective extension	10.8	19.1	**8.3**	20.1	17.0	**-3.1**	0.001	sig
CARE AND COMFORT	3.6	2.1	**-1.5**	3.26	3.23	**-0.0**	0.002	sig
GAS PT	0.0	0.81	**0.8**	0.0	0.23	**0.2**	0.002	sig
GAS T-score	0.0	59.0	**59.0**	0.0	42.7	**42.7**	0.005	sig
GMFM	34.6	37.2	**2.6**	34.7	36.2	**1.5**	0.41	
Lay & roll	76.5	76.5	**0.0**	72.1	78.4	**6.3**	>0.50	
Sit	46.5	50.2	**3.7**	47.4	48.5	**1.1**	0.13	
Crawl & kneel	24.9	26.7	**1.9**	24.3	24.9	**0.5**	0.13	
Standing	17.9	23.1	**5.1**	17.9	17.4	**-0.6**	0.017	sig
Walk, running, jump	7.4	9.7	**2.3**	11.6	11.9	**0.3**	0.006	sig
ADVERSE EFFECTS								
Sleep	2.2	2.1	**-0.1**	2.4	2.5	**0.1**	>0.50	
Bowel & bladder	3.2	2.7	**-0.5**	3.4	2.8	**-0.6**	>0.50	
Constipation	1.8	0.9	**-0.9**	1.7	1.4	**-0.2**	>0.50	
Drooling	2.3	1.8	**-0.6**	1.8	1.6	**-0.2**	>0.50	
Head control	2.0	1.6	**-0.4**	1.8	1.3	**-0.4**	>0.50	

Analysis conducted according to Wilcoxon Signed Rank Test

Note that a lower Care and Comfort and Adverse Effects score denotes improvement

QUEST = Quest-Quality of Upper Extremity Skills Test; GMFM = Gross Motor Function Measure; GAS PT = Goal Attainment Scale Physical Therapy

#### CCHQ

Parents of children in the study group reported improvement in the children's care and comfort (median change = -1.45) compared to a slight improvement in controls (median change = -0.14; Wilcoxon-signed ranked test, *p =* 0.002). Four caregivers reported deterioration in CCHQ in the control group; no deterioration was reported in the study group. Communication was the main domain that improved (*p =* 0.008) followed by personal care (*p =* 0.002).

#### GAS

Sitting, walking and fine motor manipulation skills were the main areas that were defined as goals and were individually rated. On the GAS-PT scale the study group improved significantly more than the control group (0.8 vs 0.2; *p =* 0.002). Similarly, the children who listened to gamma frequencies attained more goals (*mean change = 59*.*0*) than children who listened to music alone (mean change = 42.7; Wilcoxon-signed ranked test, *p*<0.005; Video A in S1 Video).

In all the matched pairs, study group children’s improvement exceeded that of the control group. Two thirds of the children in the study group exceed expectations (>50%), while all of the children in the control group either deteriorated (four out of nine) or remained the same (five out of nine).

#### QUEST

**Fig 2:**
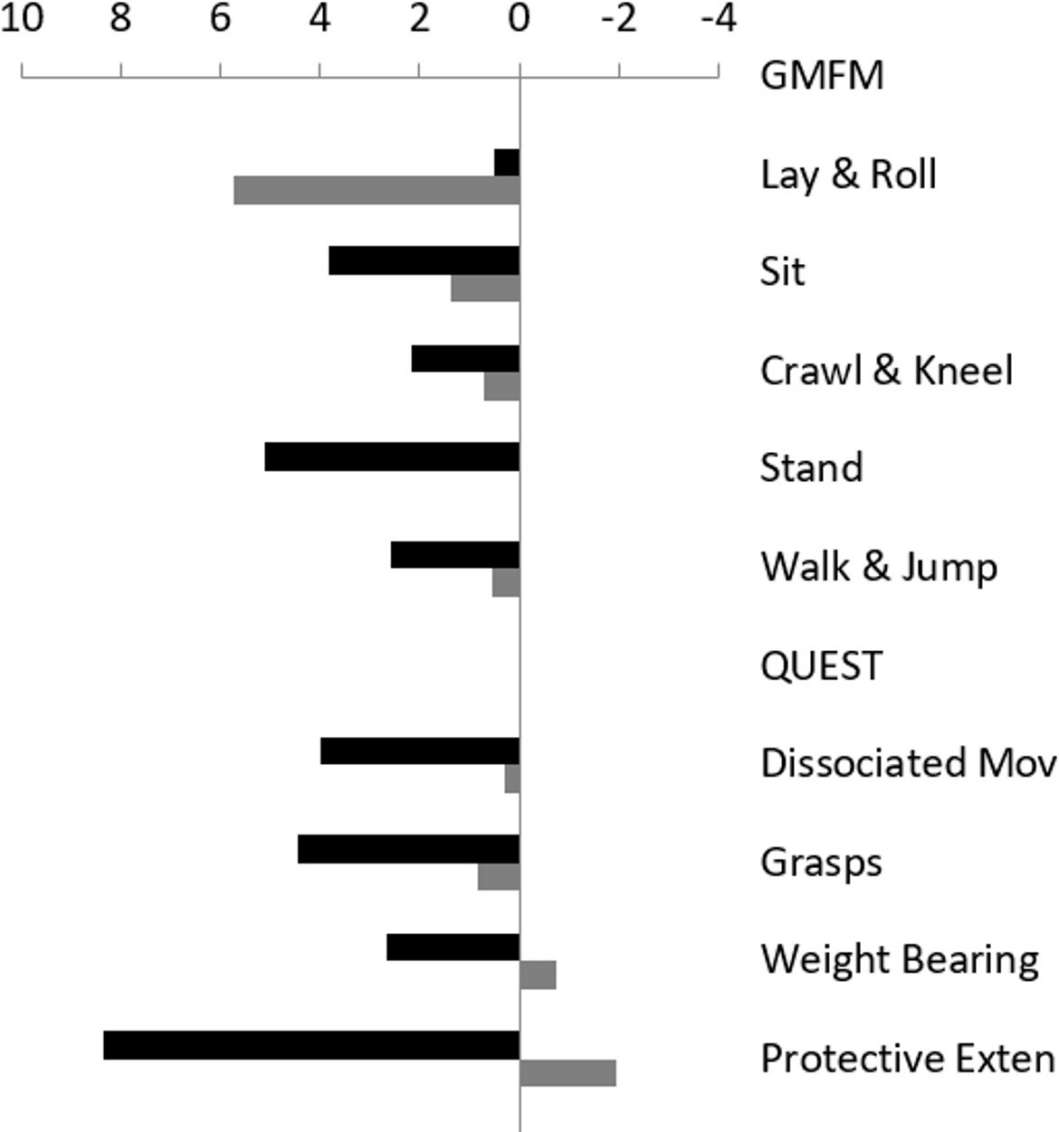
QUEST and GMFM subscales results after 5 months of auditory stimulus and sham stimulus. Children who received auditory stimuli (black bars) have improved on most motor functions both in upper limbs (associated movements, grasp, protective extension, and weight bearing) and lower limbs (sitting, crawling & kneeling, standing, and walking, running & jumping compared to controls that have not changed or deteriorated (gray bars). Lying and rolling improved in controls more than in the study group thus the total GMFM score was not significantly improved in the study group.

Children receiving gamma frequencies improved upper limb skills following the intervention (median change = 4.78) more than matched controls (mean change = -1.04; Wilcoxon-signed ranked test, *p =* 0.006; Fig 2). In the sub-dimensions of QUEST, there were significant improvements in protective extension (*p =* 0.001), weight bearing (*p =* 0.014), and grasps (*p =* 0.014).

#### GMFM

The change of the global GMFM was similar in both groups (Wilcoxon-signed ranked test; *p = 0*.*41*). In the treatment group the GMFM walking (*p =* 0.006) and standing (*p =* 0.017) scores showed significant improvement (Fig 2). In the control group there were no significant improvements in any of the sub sections.

#### Age

Age was uncorrelated with improvements in the major outcome variable scores such as QUEST, Care and Comfort, GASPT, GMFM scores. (Pearson, *r* = 0.05). Similarly, there were no significant associations between age differences of the pairs and the outcome (Spearman’s Rank order correlation *p*<0.05).

### Treatment intensity

Compliance was similar between the two groups. There were no significant differences between the treatment and control group in cumulative treatment times (386 vs 403 minutes, *p>0*.*50; Wilcoxon signed rank test*) or numbers of treatment sessions (14.1 vs 18.3, *p = 0*.*5 Wilcoxon signed rank test*). In addition, the Spearman's rank order correlation coefficient found no significant associations between cumulative treatment times or numbers of treatment sessions and outcome variables such as QUEST, Care and Comfort, GAS-PT, GMFM scores (*p*>0.05). However marginal significance was found with the GAST scores (*p =* 0.05).

Four children in each group did audio stimulation during the morning hours while the remaining children did audio stimulation at home in the late afternoon or early evening hours. Many parents reported that their child did not like wearing headphones but that compliance was best when music familiar to their child, and not nature sounds, was in the background.

### Narrative/Parental reports

Time logs were kept for all of the children. Six from the treatment group and five from the control group wrote narratives about their experience. In the study group, four parents reported improvements in their children's function, three said that their child’s body’s was more relaxed, four reported increased cognitive, attention skills, cooperation or communication skills. In the control group, two reported increased relaxation, two reported increased cooperation while three reported no change.

### Adverse effects

One parent reported that his child had more seizures following the study intervention; subsequently, this pair thus did not complete the study. Five of the 18 participating in the study were on concurrent epilepsy treatment and none reported a change in seizure frequency. There were no reports about other adverse events or difficulties to comply with the protocol assigned. Two children from the control group and two from the study group required treatments pre study for constipation (suppositories or enemas). Following intervention, the two in the control group still had constipation, though only one required treatment, while the two children in the treatment group no longer had constipation. There were no significant (*p*>0.5) adverse effects in sleep, drooling, head control, constipation, bowel and bladder.

## Discussion

This preliminary study demonstrated the potential of one month of auditory stimulation through headphones to improve functional motor skills, enhance social communications and ease caretaker burden in children with CP and hypertonia. This effect was maintained in our cohort for at least 5 months.

We suggest that the auditory stimuli had modulated neuronal circuits via the resonance phenomenon. Auditory stimulation might be used for treatment by actively enhancing neuronal oscillations using the resonance phenomenon. This phenomenon occurs when the frequency of the auditory stimulation matches a specific neuronal oscillating frequency (a preferential frequency). A preferential frequency enhances the voltage created by the membrane of the specific neurons creating the resonance phenomenon.[18] In this study, we focused on gamma frequencies (32–250 Hz) that are involved in visual, auditory, tactile, and motor processing. Pastor et al showed increased cortical synaptic activity and cerebral blood flow following a 40 Hz auditory stimulation. Edwards et al reported high gamma oscillations (60–250 Hz, centered at 100 Hz) from left temporal areas in response to auditory tone stimuli. Various low frequency sensory stimulations, including sound up to 30 Hz, could have physiological effects, on specific neurological disorders.[8] Motor cortical stimulation amplifies and synchronizes oscillatory neural activity associated with motor function.[19] In our study, we found improvement in motor skills suggesting that therapeutic effects could be linked to the various mechanisms described in this discussion.

Brain oscillatory activity is also closely linked to GABA-mediated motor neuron inhibition and GABA agonists are effective in treating spasticity.[20,21] Increased gamma oscillations may amplify the GABAergic inhibitory neurotransmission, reducing hypertonicity similar to other neuromodulation techniques such as rTMS.[22] Increased GABA inhibition following auditory stimulation may explain the significant relaxation, and easier caretaking activities such as dressing, bathing, transferring and feeding their children.

Auditory stimulation improved communication as shown in the CCHQ. In addition, a number of parents of children in the study group who claimed that pretreatment their children cried constantly and were uncooperative, reported that post treatment, their children were calmer and more cooperative. Interestingly, the CCHQ question that was most sensitive to intervention was “How easy is it for your child to be completely understood by those who know your child well?”Gamma oscillations are also closely related to sensory integration, communication and speech.[23] We postulate that auditory stimulation improves synchronicity, producing the accompanying benefits in communication, behavior, cooperation and mood.

Frequency modulated and amplitude modulated tones have been hypothesized to generate considerably greater activity in brain regions beyond the auditory areas as compared to un-modulated tones, this could explain the benefits achieved by the children in the treatment group.[24] Interestingly, benefits resulting from the auditory stimulation were sustained long after the cessation of the one month treatment period, that could be attributed to synaptic neuro-plasticity and network connectivity modification.[25] We postulate that this neuroplasticity creates the long term benefits lasting at least 5 months. Functional improvements in children with CP are the main parameter for intervention efficacy in CP.[26] Many interventions that are commonly used in children with CP have not demonstrated to have a functional impact.[3] Thus, auditory stimulation is one of the few noninvasive safe interventions that has a functional impact on daily life of the child with CP and their families. There were no reported adverse effects except that one child was reported to have more seizures. We consider this incidental and do not attribute this to treatment since many children in the treatment group had epilepsy and none reported an increase in seizures.

Limitations of the study: small study size. We did not assess emotional impact and quality of life. Demographic variables were not covaried because of the small sample and the use of non-parametric tests.

## Conclusion

Children with CP and hypertonia might have functional improvement following auditory stimuli. This safe, non-invasive novel alternative intervention may provide an opportunity for children with CP to improve their motor skills and increase their communication skills in their home, school and community while at the same time reducing the burden of the caretakers.

## Supporting information

S1 Questionnaire Adverse effects. Bowel and bladder control (5-point scale), sleep, drooling and head control (4-point scale) and constipation (3-point scale) at baseline and after 5 months.
(DOCX)

S1 Table Raw data. Data of all participants before and after intervention.
(PDF)

S1 Video Gross motor skills before and after treatment. Three children in the treatment group who gained motor skills during the study. The motor skills shown are Sit to Stand, Standing and Walking. The videos were filmed before treatment and after 5 months.
(MP4)

## Abbreviations

CCHQ: Care and Comfort Hypertonicity Questionnaire
GAS: Goal Attainment Scale
GMFCS: Gross Motor Functional Classification Scale
GMFM: Gross Motor Function Measure
MACS: Manual Ability Classification System
QUEST: Quest-Quality of upper extremity skills test

## References

[1] Hagberg B, Hagberg G, Olow I, von Wendt L (1996) The changing panorama of cerebral palsy in Sweden. VII. Prevalence and origin in the birth year period 1987–90. Acta Paediatr 85: 954–960. 8863878 10.1111/j.1651-2227.1996.tb14193.x

[2] Mihaylov SI, Jarvis SN, Colver AF, Beresford B (2004) Identification and description of environmental factors that influence participation of children with cerebral palsy. Dev Med Child Neurol 46: 299–304. 15132259 10.1017/s0012162204000490

[3] Novak I, McIntyre S, Morgan C, Campbell L, Dark L, Morton N, et al (2013) A systematic review of interventions for children with cerebral palsy: state of the evidence. Dev Med Child Neurol 55: 885–910. 10.1111/dmcn.12246 23962350 10.1111/dmcn.12246

[4] Kirton A, Chen R, Friefeld S, Gunraj C, Pontigon AM, Deveber G. (2008) Contralesional repetitive transcranial magnetic stimulation for chronic hemiparesis in subcortical paediatric stroke: a randomised trial. Lancet Neurol 7: 507–513. 10.1016/S1474-4422(08)70096-6 18455961 10.1016/S1474-4422(08)70096-6

[5] Aree-uea B, Auvichayapat N, Janyacharoen T, Siritaratiwat W, Amatachaya A, Prasertnoo J et al (2014) Reduction of spasticity in cerebral palsy by anodal transcranial direct current stimulation. J Med Assoc Thai 97: 954–962. 25536713

[6] Koy A, Hellmich M, Pauls KA, Marks W, Lin JP, Fricke O, et al (2013) Effects of deep brain stimulation in dyskinetic cerebral palsy: a meta-analysis. Mov Disord 28: 647–654. 10.1002/mds.25339 23408442 10.1002/mds.25339

[7] Keen JR, Przekop A, Olaya JE, Zouros A, Hsu FP (2014) Deep brain stimulation for the treatment of childhood dystonic cerebral palsy. J Neurosurg Pediatr 14: 585–593. 10.3171/2014.8.PEDS141 25325412 10.3171/2014.8.PEDS141

[8] Salansky N, Fedotchev A, Bondar A (1998) Responses of the nervous system to low frequency stimulation and EEG rhythms: clinical implications. Neurosci Biobehav Rev 22: 395–409. 9579328 10.1016/s0149-7634(97)00029-8

[9] Hwang M, Kuroda MM, Tann B, Gaebler-Spira DJ (2011) Measuring care and comfort in children with cerebral palsy: the care and comfort caregiver questionnaire. PM R 3: 912–919. 10.1016/j.pmrj.2011.05.017 21852220 10.1016/j.pmrj.2011.05.017

[10] Baker KW, Tann B, Mutlu A, Gaebler-Spira D (2014) Improvements in children with cerebral palsy following intrathecal baclofen: use of the Rehabilitation Institute of Chicago Care and Comfort Caregiver Questionnaire (RIC CareQ). J Child Neurol 29: 312–317. 10.1177/0883073812475156 23420651 10.1177/0883073812475156

[11] Maloney FP, Mirrett P, Brooks C, Johannes K (1978) Use of the Goal Attainment Scale in the treatment and ongoing evaluation of neurologically handicapped children. Am J Occup Ther 32: 505–510. 151501

[12] Cusick A, McIntyre S, Novak I, Lannin N, Lowe K (2006) A comparison of goal attainment scaling and the Canadian Occupational Performance Measure for paediatric rehabilitation research. Pediatr Rehabil 9: 149–157. 10.1080/13638490500235581 16449074 10.1080/13638490500235581

[13] Kiresuk TJ, Sherman RE (1968) Goal attainment scaling: A general method for evaluating comprehensive community mental health programs. Community Ment Health J 4: 443–453. 10.1007/BF01530764 24185570 10.1007/BF01530764

[14] Turner-Stokes L (2009) Goal attainment scaling (GAS) in rehabilitation: a practical guide. Clin Rehabil 23: 362–370. 10.1177/0269215508101742 19179355 10.1177/0269215508101742

[15] Dodd KJ, Taylor NF, Graham HK (2003) A randomized clinical trial of strength training in young people with cerebral palsy. Dev Med Child Neurol 45: 652–657. 14515935 10.1017/s0012162203001221

[16] Damiano DL, Abel MF (1998) Functional outcomes of strength training in spastic cerebral palsy. Arch Phys Med Rehabil 79: 119–125. 9473991 10.1016/s0003-9993(98)90287-8

[17] Haga N, van der Heijden-Maessen HC, van Hoorn JF, Boonstra AM, Hadders-Algra M (2007) Test-retest and inter- and intrareliability of the quality of the upper-extremity skills test in preschool-age children with cerebral palsy. Arch Phys Med Rehabil 88: 1686–1689. 10.1016/j.apmr.2007.07.030 18047886 10.1016/j.apmr.2007.07.030

[18] Matsumoto-Makidono Y, Nakayama H, Yamasaki M, Miyazaki T, Kobayashi K, Wanatanabe M, et al (2016) Ionic Basis for Membrane Potential Resonance in Neurons of the Inferior Olive. Cell Rep 16: 994–1004. 10.1016/j.celrep.2016.06.053 27425615 10.1016/j.celrep.2016.06.053

[19] Joundi RA, Jenkinson N, Brittain JS, Aziz TZ, Brown P (2012) Driving oscillatory activity in the human cortex enhances motor performance. Curr Biol 22: 403–407. 10.1016/j.cub.2012.01.024 22305755 10.1016/j.cub.2012.01.024 PMC3343257

[20] Buzsáki G, Kaila K, Raichle M (2007) Inhibition and Brain Work. Neuron 56: 771–783. 10.1016/j.neuron.2007.11.008 18054855 10.1016/j.neuron.2007.11.008 PMC2266612

[21] van Doornik J, Kukke S, McGill K, Rose J, Sherman-Levine S, Sanger TD. (2008) Oral baclofen increases maximal voluntary neuromuscular activation of ankle plantar flexors in children with spasticity due to cerebral palsy. J Child Neurol 23: 635–639. 10.1177/0883073807313046 18281622 10.1177/0883073807313046

[22] Kirton A, Andersen J, Herrero M, Nettel-Aguirre A, Carsolio L, Keess J et al (2016) Brain stimulation and constraint for perinatal stroke hemiparesis: The PLASTIC CHAMPS Trial. Neurology 86: 1659–1667. 10.1212/WNL.0000000000002646 27029628 10.1212/WNL.0000000000002646 PMC4854585

[23] Kingyon J, Behroozmand R, Kelley R, Oya H, Kawasaki H, Narayanan NS, et al (2015) High-gamma band fronto-temporal coherence as a measure of functional connectivity in speech motor control. Neuroscience 305: 15–25. 10.1016/j.neuroscience.2015.07.069 26232713 10.1016/j.neuroscience.2015.07.069 PMC4747053

[24] Hart HC, Palmer AR, Hall DA (2003) Amplitude and frequency-modulated stimuli activate common regions of human auditory cortex. Cereb Cortex 13: 773–781. 12816893 10.1093/cercor/13.7.773

[25] Buzsaki G (2006) Rhythms of the Brain Oxford University Press.

[26] Rosenbaum P, Eliasson AC, Hidecker MJ, Palisano RJ (2014) Classification in childhood disability: focusing on function in the 21st century. J Child Neurol 29: 1036–1045. 10.1177/0883073814533008 24810083 10.1177/0883073814533008

