# Supplementary material for: Auditory stimulation improves motor function and caretaker burden in children with cerebral palsy- A randomized double blind study
Source: PLoS One. 2018 Dec 13;13(12):e0208792. doi: 10.1371/journal.pone.0208792 (PMC6292588; doi:10.1371/journal.pone.0208792)
Supplement: S1 Questionnaire — Bowel and bladder control (5-point scale), sleep, drooling and head control (4-point scale) and constipation (3-point scale) at baseline and after 5 months. (DOCX) [file pone.0208792.s001.docx]

**Supplementary Questionnaire**

1. During the last week, was it difficult for your child to fall asleep at night?

**A** not at all difficult **B** mild difficultly **C** moderate difficulty **D** very difficult

1. During the last week, how many times, on the average, did your child wake up during the night?

**A** did not wake at all **B** one time **C** two to three times **D** more than three times

1. During the last week, how many hours, on the average, did your child sleep at night?

**A** more than 12 hours **B** 11-12 hours **C** 10-11 hours **D** 9-10 hours

**E** less than 9 hours

1. During the last week, did your child have a loss of bladder control?

**A** no **B** Yes,1 time **C** Yes. 2-3 times **D** Yes. more than 3 times **E** my child has no control

1. During the last week, did your child have a loss of bowel control?

**A** no **B** Yes,1 time **C** Yes. 2-3 times **D** Yes. more than 3 times **E** my child has no control

1. During the last week, did your child suffer from constipation?

**A** no **B** Yes, but does not require treatment **C** Yes, requires suppositories/laxatives

**D** Yes, requires enemas.

1. During the last week, did your child drool?

**A** not at all **B** Yes, mild drooling **C** Yes, moderate drooling **D** Yes, severe drooling

1. During the last week, did your child have difficulty with head control?

**A** not at all **B** Yes, mild difficulty **C** Yes, moderate difficulty **D** Yes, severe difficulty
