# Supplementary material for: Auditory stimulation improves motor function and caretaker burden in children with cerebral palsy- A randomized double blind study
Source: PLoS One. 2018 Dec 13;13(12):e0208792. doi: 10.1371/journal.pone.0208792 (PMC6292588; doi:10.1371/journal.pone.0208792)
Supplement: S1 Table — Data of all participants before and after intervention. (PDF) [file pone.0208792.s002.pdf]

child #	Group (cc	age (Year	School (3	Gender (I	Cognitive	Verbal (y	anatomic	Spasticity
B1	1.00	7.20	2.00	1.00	1.00	0.00	1.00	0.00
B3	1.00	3.20	2.00	1.00	3.00	1.00	2.00	1.00
B6	1.00	2.90	2.00	1.00	3.00	1.00	1.00	1.00
C1	1.00	17.00	3.00	1.00	2.50	1.00	1.00	1.00
C5	1.00	7.50	3.00	1.00	2.50	1.00	2.00	1.00
D1	1.00	5.30	4.00	0.00	2.00	0.00	1.00	1.00
D3	1.00	5.70	4.00	1.00	1.00	0.00	1.00	1.00
D5	1.00	10.00	4.00	1.00	3.00	1.00	2.00	1.00
D8	1.00	11.00	4.00	1.00	1.50	1.00	0.00	1.00
B2	0.00	6.60	2.00	1.00	2.50	0.00	1.00	1.00
B4	0.00	3.30	2.00	1.00	3.00	1.00	2.00	1.00
B5	0.00	2.40	2.00	0.00	3.00	0.00	1.00	1.00
C2	0.00	16.00	3.00	1.00	2.50	1.00	1.00	1.00
C6	0.00	8.00	3.00	1.00	2.50	1.00	1.00	1.00
D2	0.00	6.30	4.00	0.00	2.00	0.00	1.00	1.00
D4	0.00	4.70	4.00	1.00	1.00	0.00	1.00	1.00
D6	0.00	9.10	4.00	0.00	3.00	0.00	2.00	1.00
D7	0.00	8.00	4.00	0.00	1.50	1.00	0.00	1.00

blue= baseline
green= at 5 months (5mo)
italics= subitems

Dystonia	GMFCS (=	MACS (=M	GMFM (=	QUEST = Que	CCHQ (=C	GAS (=Goal a	GMFM_5n	QUEST_5
1.00	5.00	5.00	3.35	-4.48	2.98	0.00	3.35	-7.60
0.00	2.50	1.00	68.63	89.72	2.58	0.00	73.13	89.86
1.00	4.00	3.00	26.47	43.91	4.50	0.00	43.40	62.80
1.00	5.00	4.00	28.57	14.19	3.48	0.00	26.20	16.20
0.00	3.00	3.00	69.20	70.91	1.71	0.00	69.16	69.67
1.00	5.00	5.00	15.22	0.30	4.99	0.00	15.49	6.06
1.00	5.00	5.00	17.63	-0.23	4.59	0.00	19.50	7.20
0.00	4.00	4.00	25.82	14.41	4.04	0.00	31.82	21.34
0.00	3.00	3.00	56.84	35.63	3.54	0.00	53.06	41.88
1.00	5.00	5.00	19.29	2.01	3.22	0.00	20.21	2.16
0.00	2.00	1.00	86.70	87.40	1.48	0.00	88.28	81.89
1.00	4.00	3.00	14.24	17.28	3.13	0.00	30.07	14.96
1.00	5.00	4.00	25.51	20.48	3.66	0.00	25.69	17.56
0.00	3.00	3.00	66.00	72.54	3.60	0.00	64.14	70.33
1.00	5.00	4.00	17.80	21.18	2.80	0.00	16.12	18.29
1.00	5.00	5.00	12.02	0.06	5.50	0.00	14.34	0.06
1.00	4.00	4.00	33.22	27.66	2.72	0.00	32.92	31.32
1.00	3.00	3.00	37.32	47.23	3.19	0.00	34.16	49.94

CCHQ_5m	GAS PT 5	GAS_Tsco	*Lyingand*	*Sitting GM*	*Crawland*	*Standing*	*WalkrunJ*	*Lyingand*
2.30	1.00	77.39	*11.76*	*5.00*	*0.00*	*0.00*	*0.00*	*11.76*
1.13	1.00	60.88	*96.08*	*98.33*	*83.33*	*48.72*	*16.67*	*98.04*
2.55	0.92	50.00	*84.31*	*38.33*	*7.14*	*2.56*	*0.00*	*92.16*
2.81	0.50	45.44	*84.31*	*41.67*	*14.29*	*2.56*	*0.00*	*80.39*
0.00	0.88	60.88	*96.08*	*96.67*	*78.57*	*53.85*	*20.83*	*96.08*
1.18	0.83	68.26	*62.75*	*13.33*	*0.00*	*0.00*	*0.00*	*60.78*
2.85	1.00	59.13	*76.47*	*11.67*	*0.00*	*0.00*	*0.00*	*78.43*
3.56	0.75	63.69	*88.24*	*21.67*	*7.14*	*5.13*	*6.94*	*88.24*
2.40	0.42	45.44	*88.24*	*91.67*	*33.33*	*48.72*	*22.22*	*82.35*
3.02	0.25	50.00	*64.71*	*25.00*	*0.00*	*2.56*	*4.17*	*70.59*
1.65	0.50	50.00	*94.12*	*100.00*	*95.24*	*87.18*	*56.94*	*100.00*
2.88	0.13	50.00	*35.29*	*33.33*	*0.00*	*2.56*	*0.00*	*94.12*
3.29	0.25	39.12	*80.39*	*40.00*	*7.14*	*0.00*	*0.00*	*80.39*
3.69	0.33	50.00	*96.08*	*85.00*	*83.33*	*46.15*	*19.44*	*94.12*
2.57	0.00	22.61	*56.86*	*25.00*	*7.14*	*0.00*	*0.00*	*54.90*
6.08	0.00	31.74	*45.10*	*15.00*	*0.00*	*0.00*	*0.00*	*50.98*
2.86	0.38	50.00	*90.20*	*48.33*	*16.67*	*2.56*	*8.33*	*88.24*
3.05	0.25	40.87	*86.27*	*55.00*	*9.52*	*20.51*	*15.28*	*72.55*

Sitting_5r	Crawland	Standing_	WalkrunJ	# of sessi	treatmen	Dissociat	Grasps Ql	WeightBe
5.00	0.00	0.00	0.00	16.00	496.00	12.50	-25.93	0.00
95.00	78.57	71.79	22.22	25.00	500.00	95.24	73.91	100.00
58.33	30.95	23.08	12.50	4.00	46.00	70.31	33.33	72.00
38.33	7.14	5.13	0.00	15.00	450.00	35.94	14.81	6.00
96.67	80.95	51.28	20.83	15.00	450.00	87.50	48.15	98.00
16.67	0.00	0.00	0.00	27.00	800.00	23.44	-22.22	0.00
16.67	2.38	0.00	0.00	4.00	100.00	25.00	-25.93	0.00
40.00	7.14	15.38	8.33	12.00	360.00	43.75	0.00	0.00
85.00	33.33	41.03	23.61	9.00	270.00	54.69	18.52	36.00
23.33	7.14	0.00	0.00	11.00	261.00	26.56	-18.52	0.00
100.00	95.24	79.49	66.67	25.00	351.00	92.19	74.07	100.00
40.00	9.52	2.56	4.17	2.00	20.00	46.67	-14.81	20.00
38.33	7.14	2.56	0.00	15.00	410.00	50.00	14.81	6.00
85.00	78.57	43.59	19.44	15.00	450.00	76.56	70.37	96.00
23.33	2.38	0.00	0.00	20.00	535.00	45.31	7.41	32.00
18.33	2.38	0.00	0.00	22.00	414.00	18.75	-18.52	0.00
50.00	14.29	5.13	6.94	10.00	300.00	46.88	11.11	36.00
58.33	7.14	23.08	9.72	45.00	890.00	53.13	55.56	58.00

Protective	*Dissociat*	*Grasps QI*	*WeightBe*	*Protective*	sleep- Ad	bowelanc	constipat	drooling-
0.00	*3.13*	*-25.93*	*0.00*	*0.00*	2.33	5.00	2.00	3.00
0.00	*100.00*	*69.57*	*100.00*	*0.00*	3.33	5.00	1.00	1.00
0.00	*68.75*	*44.44*	*88.00*	*50.00*	3.00	5.00	1.00	3.00
0.00	*50.00*	*14.81*	*0.00*	*0.00*	2.33	1.00	2.00	1.00
50.00	*82.81*	*62.96*	*94.00*	*38.89*	1.00	3.00	1.00	1.00
0.00	*39.06*	*-14.81*	*0.00*	*0.00*	2.33	1.50	3.00	3.00
0.00	*34.38*	*-22.22*	*0.00*	*16.67*	2.00	5.00	3.00	3.00
13.89	*51.56*	*11.11*	*6.00*	*16.67*	1.67	1.00	1.00	3.00
33.33	*54.69*	*14.81*	*48.00*	*50.00*	2.00	2.50	2.00	3.00
0.00	*23.44*	*-14.81*	*0.00*	*0.00*	2.00	5.00	1.00	3.00
83.33	*81.25*	*74.07*	*100.00*	*72.22*	3.00	5.00	1.00	1.00
0.00	*30.00*	*-11.11*	*26.00*	*0.00*	2.00		1.00	1.00
11.11	*53.13*	*11.11*	*6.00*	*0.00*	2.00	1.50	3.00	1.00
47.22	*84.38*	*70.37*	*96.00*	*30.56*	3.00	2.50	1.00	1.00
0.00	*43.75*	*7.41*	*22.00*	*0.00*	2.00	5.00	1.00	3.00
0.00	*18.75*	*-18.52*	*0.00*	*0.00*	3.67	5.00	4.00	4.00
16.67	*57.81*	*14.81*	*36.00*	*16.67*	2.00	1.00	1.00	1.00
22.22	*62.50*	*51.85*	*52.08*	*33.33*	2.00	2.00	2.00	1.00

headcont	sleep- Ad	bowelanc	constipat	drooling-	headcontrol- Adverse effects item
4.00	1.67	5.00	1.00	2.00	3.00
1.00	2.33	5.00	1.00	1.00	1.00
2.00	2.67	5.00	1.00	2.00	2.00
2.00	2.67	2.00	2.00	1.00	1.00
1.00	1.67	3.00	1.00	1.00	1.00
4.00	1.33	0.00	0.00	2.00	4.00
1.00	2.67	2.00	0.00	3.00	0.00
1.00	1.67	1.00	1.00	2.00	1.00
1.00	2.67	1.50	1.00	2.00	1.00
1.00	2.00	5.00	1.00	3.00	1.00
1.00	3.33	2.00	1.00	1.00	1.00
1.00	2.00		1.00	1.00	1.00
1.00	2.00	1.00	2.00	1.00	1.00
1.00	3.00	2.50	1.00	1.00	1.00
4.00	2.33	5.00	1.00	2.00	3.00
4.00	3.67	5.00	4.00	4.00	1.00
2.00	2.00	1.50	2.00	1.00	3.00
1.00	2.00	0.00	0.00	0.00	0.00
